# Construct prediction models for low muscle mass with metabolic syndrome using machine learning

**DOI:** 10.1371/journal.pone.0331925

**Published:** 2025-09-09

**Authors:** Yanxuan Wu, Fu Li, Hao Chen, Liang Shi, Meng Yin, Fan Hu, Gongchang Yu

**Affiliations:** 1 Neck-Shoulder and Lumbocrural Pain Hospital of Shandong First Medical University, Shandong First Medical University & Shandong Academy of Medical Sciences, Jinan, China; 2 Shandong First Medical University & Shandong Academy of Medical Sciences, Jinan, China; UTMB: The University of Texas Medical Branch at Galveston, UNITED STATES OF AMERICA

## Abstract

**Background:**

Metabolic syndrome (MetS) and sarcopenia are major global public health problems, and their coexistence significantly increases the risk of death. In recent years, this trend has become increasingly prominent in younger populations, posing a major public health challenge. Numerous studies have regarded reduced muscle mass as a reliable indicator for identifying pre-sarcopenia. Nevertheless, there are currently no well-developed methods for identifying low muscle mass in individuals with MetS.

**Methods:**

A total of 2,467 MetS patients (aged 18–59 years) with low muscle mass assessed by dual-energy X-ray absorptiometry (DXA) were included using data from the 2011–2018 National Health and Nutrition Examination Survey (NHANES). Least Absolute Shrinkage and Selection Operator (LASSO) regression was then used to screen for important features. A total of nine Machine learning (ML) models were constructed in this study. Area under the curve (AUC), F1 Score, Recall, Precision, Accuracy, Specificity, PPV, and NPV were used to evaluate the model’s performance and explain important predictors using the Shapley Additive Explain (SHAP) values.

**Results:**

The Logistic Regression (LR) model performed the best overall, with an AUC of 0.925 (95% CI: 0.9043, 0.9443), alongside strong F1-score (0.87) and specificity (0.89). Five important predictors are displayed in the summary plot of SHAP values: height, gender, waist circumference, thigh length, and alkaline phosphatase (ALP).

**Conclusion:**

This study developed an interpretable ML model based on SHAP methodology to identify risk factors for low muscle mass in a young population of MetS patients. Additionally, a web-based tool was implemented to facilitate sarcopenia screening.

## Introduction

Metabolic syndrome (MetS) is characterized by a cluster of metabolic risk factors, including central obesity, hyperglycemia, dyslipidemia, and elevated blood pressure, driven by shared pathophysiological pathways [1]. This is a contributing factor to type 2 diabetes, cardiovascular disease, and other adverse health outcomes [2]. Although the prevalence of MetS is higher in older populations, in recent years it has become increasingly common in young people worldwide, especially in those who are obese, sedentary, and have unhealthy diets [3]. The prevalence of MetS increased significantly between 2011 and 2018, according to the MetS prevalence trends for U.S. adults study [4]. Patients with MetS often suffer from a loss of muscle mass, which can lead to sarcopenia, further affecting the patient’s quality of life and health prognosis [5]. Sarcopenia is a skeletal muscle disease characterized by decreased muscle mass and strength and decreased physical function [6]. It was primarily thought that sarcopenia was a disease associated with the older population. However, it is worth noting that the incidence of sarcopenia has shown a clear trend towards a younger population [7]. This may be closely related to changes in modern lifestyles, such as prolonged sedentary lifestyles and lack of physical activity, which accelerate the loss of skeletal muscle and increase the risk of the disease in the younger population. A study from the China Health and Retirement Longitudinal Study (CHARLS) showed that adults with sarcopenia had a 41% elevated 7-year risk of death compared to a non-patient population [8].

MetS and sarcopenia are major public health problems worldwide. MetS has been demonstrated to induce persistent inflammation, oxidative stress, and fat accumulation, thereby contributing to a pathological state of skeletal muscle [9]. MetS and its components, central obesity and hypertension, significantly increase the prevalence of sarcopenia in both men and women compared to individuals without MetS [10].

The coexistence of MetS and sarcopenia has been shown to increase the risk of mortality from any cause and mortality from specific causes, particularly in non-elderly populations, as evidenced by longitudinal analysis [11]. A growing amount of studies has demonstrated a bidirectional association between MetS and sarcopenia [10,12]. One of the most important pieces of evidence is insulin resistance (IR) [13]. IR is a central driver of MetS [14]. Insulin-regulated skeletal muscle is the most important tissue for the uptake of glucose, and their uptake accounts for 80% of systemic insulin-mediated glucose uptake [15]. IR makes muscle cells less responsive to insulin [16]. This leads to less efficient glucose uptake and utilization, impaired energy metabolism, and impairment of normal muscle function. On the one hand, IR is an important pathogenetic mechanism that induces muscle decay [17]. On the other hand, sarcopenia has been shown to induce the development of insulin resistance through a variety of mechanisms, including endoplasmic reticulum stress, abnormal lipid infiltration, and other pathways, ultimately resulting in a vicious cycle [18].

MetS is closely related to muscle strength [19]. The fundamental element underpinning this association is a decline in skeletal muscle mass, a phenomenon that is particularly evident in patients exhibiting skeletal sarcopenia [20]. Levels of grip strength, a significant metric for evaluating muscle strength, have been demonstrated to exhibit a negative correlation with the prevalence of MetS [21]. More seriously, studies have noted that decreased grip strength in adults is associated with an increased risk of all-cause mortality [22].

Dual-energy X-ray absorptiometry (DXA) is one of the most widely used non-invasive tools for the assessment of sarcopenia [23]. However, DXA equipment is expensive and not portable, making it difficult to be widely used in large sample studies and clinical work testing. Current evidence suggests to be that it is much easier to mitigate muscle mass decline than to rescue muscle mass that has been lost [24]. Even in clinical trials, there isn’t a recognized medication regimen for treating sarcopenia at the moment [25]. In individuals with MetS, early detection of skeletal sarcopenia is critical. The early symptoms of sarcopenia are usually not obvious, and many patients are not diagnosed until they are experiencing functional limitations and a loss of strength [26]. By focusing on a younger age group, our study aimed to capture early muscle changes at a stage when timely intervention may still prevent progression to overt sarcopenia.

Machine learning (ML) has shown great potential for applications in medicine, especially in the early prediction of chronic diseases [27]. A non-invasive model has been proposed to predict pre-sarcopenia, using low muscle mass as its indicator, in patients with stroke and non-alcoholic fatty liver disease [28,29]. The coexistence of MetS and sarcopenia has received increasing attention, but there is still a research gap on the complex interaction mechanisms and early risk prediction. In this context, our study developed a machine learning model to identify risk factors associated with low muscle mass in individuals with MetS, based on data from the National Health and Nutrition Examination Survey (NHANES). Importantly, reduced muscle mass is recognized as the most fundamental and quantifiable characteristic of sarcopenia, especially in its early stages. Several studies have used low muscle mass as a defining criterion for sarcopenia [30,31]. Accordingly, our study focuses on predicting low muscle mass to support early screening efforts before clinical diagnosis of sarcopenia becomes evident.

## Materials and methods

### Data source

Data for this study came from NHANES (https://wwwn.cdc.gov/nchs/nhanes/default.aspx). This is a population-based cross-sectional survey study, a national research project led by the National Center for Health Statistics (NCHS) to assess the health and nutritional status of adults and children in the United States. There was no need for an extra ethical assessment by the investigator’s affiliations because the work was based on publicly available deidentified data. The data were analyzed anonymously. NHANES was approved by the NCHS Research Ethics Review Board, and the study protocol adhered to the ethical guidelines set forth in the 1975 Declaration of Helsinki. All those enrolled provided written informed consent to participate.

**Fig 1:**
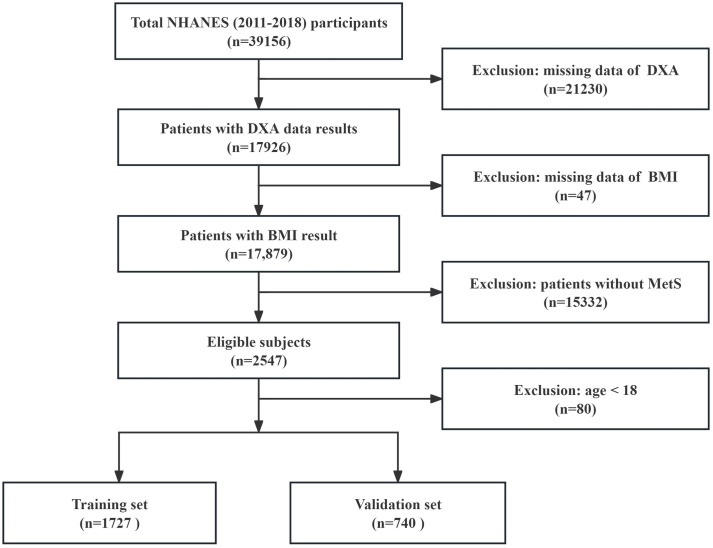
The overview of study design and patient selection flowchart.

The study’s participants were selected from NHANES who were undergoing DXA treatment between 2011 and 2018 and had a MetS diagnosis. Above all, the age range of patients included in this study was restricted to 18–59 years old in order to ensure data consistency, as people undergoing whole-body DXA in NHANES were 60 years of age and younger. This approach is consistent with sarcopenia studies based on NHANES data [32]. Detailed subject screening procedures are shown in Fig 1.

### Measurements and definition

At least three of the following criteria must be satisfied for a diagnosis of MetS, according to a statement from the American Heart Association and National Heart, Lung, and Blood Institute [33]: (1) Central obesity: Waist circumference ≥ 102 cm in men and waist circumference ≥ 88 cm in women. (2) Hyperglycemia: taking medication for high blood sugar or having an FPG of 5.6 mmol/L (100 mg/dL). (3) Hypertriglyceridemia: TG ≥ 1.7 mmol/L (150 mg/dL) or receiving treatment for elevated TG. (4) Hypertension: systolic blood pressure≥130 mmHg and/or diastolic blood pressure ≥85 mmHg or receiving antihypertensive medication. (5) Dyslipidemia: HDL < 1.0 mmol/L (40 mg/dL) in men and < 1.3 mmol/L (50 mg/dL) in women, or medication therapy to lower HDL. NHANES used the Hologic Discovery model A densitometers (Hologic, Inc., Bedford, Massachusetts) to perform a DXA whole-body scan to measure body composition. Individuals were excluded from DXA screening if they were pregnant, weighed more than 450 lb (136 kg), or were taller than 6 ft 5 in (196 cm).

Low muscle mass is a central feature of sarcopenia. Low muscle mass is measured using a Foundation for the National Institutes of Health (FNIH) recognized metric, the Skeletal Muscle Mass Index (SMI) [34]. The BMI-normalized appendicular skeletal muscle mass (ASM) is then employed, thereby obviating disparities in the effects of individual body size [24]. ASM is the total mass of skeletal muscle contained in the extremity portion of the body. ASM is widely used in sarcopenia studies as an important indicator to assess skeletal muscle health and is often used to measure loss of muscle mass. Women with an SMI <0.512 and men with an SMI <0.789 were considered to have lower muscle mass [30].

Race was categorized as Mexican American, non-Hispanic white, non-Hispanic black, other Hispanic, and other race. Education level is divided into three levels: below high school, high school, and above high school. The poverty income ratio (PIR) is the ratio of household income to the poverty line. BMI is weight divided by height squared, usually expressed in kg/m². Smoking group is categorized as current smoker, former smoker, and never smoker. A person was classified as diabetic if he or she self-reported diabetes or had a fasting blood glucose level of 126 mg/dL or higher or a glycosylated hemoglobin level of 6.5 percent or higher [35]. Systolic and diastolic blood pressures were averages of three readings.

### Feature selection

The literature on MetS and sarcopenia was reviewed, and characteristics were collected, including demographics, clinical data, laboratory tests, and self-report questionnaires. In line with previous studies that developed sarcopenia prediction models using ML, and to ensure the practicality and clinical applicability of our model, we focused on commonly used anthropometric indicators and readily available laboratory tests [36]. Feature columns with a proportion of missing values greater than 30% in the dataset were removed. Variables with a proportion of missing values less than 30% were supplemented using multiple interpolation. Least Absolute Shrinkage and Selection Operator (LASSO) regression was then used to screen for important features. LASSO regression is a variation of linear regression that accomplishes feature selection by scaling the regression coefficients of less influential predictors to zero, thereby removing them from the model. By introducing the regularization parameter lambda, LASSO regression controls the complexity of the model. Subsequently, we refined the LASSO model through 5-fold cross-validation and determined Lambda.1se as the regularization parameter for the final model. This is the largest λ value whose cross-validated error remains within one standard error of the minimum. It choice balances model complexity and generalizability, helping to reduce overfitting by selecting fewer but more robust predictors. It is based on the principle of parsimony, as lambda.1se typically yields a sparser model with slightly lower but more stable performance compared to lambda.min.

### Model construction

A total of nine ML models were constructed in this study, namely Logistic Regression (LR), Support Vector Machine (SVM), Random Forest (RF), Extreme Gradient Boosting (XGB), Light Gradient Boosting Machine (LGBM), Gradient Boosting Decision Tree (GBDT), Multi-layer Perceptron (MLP), Decision Tree (DT), CatBoost. All data included in the analysis were from four cycles of NHANE 2011–2018. The subject dataset was randomly divided into a 70% training set for model training and a 30% internal validation set for model evaluation.

To determine the optimal hyperparameters for the ML models, each model was hyperparametrically tuned using a grid search, 5-fold cross validation. The test set was not used during the model tuning phase and was only used to complete model selection and model evaluation after training. The training set was pre-processed by applying the synthetic minority over-sampling technique (SMOTE) to address the problem of positive and negative sample imbalance. Benchmarking is a systematic method for evaluating and comparing the performance of different ML models. It ensures fair and objective comparisons by running multiple models on standardized data sets and using consistent evaluation metrics. To comprehensively evaluate model performance, we selected the following metrics: area under the curve of the receiver operating characteristics (AUC-ROC), F1 value, recall, precision, accuracy, specificity, positive predictive value (PPV), and negative predictive value (NPV). Bootstrap resampling was then used to calculate 95% confidence intervals for the AUC values of each model.

In this study, to identify the most important risk factors and to explain the contribution of each characteristic to the prediction, Shapley Additive explanations (SHAP) are used. The core idea is to explain the model’s output by calculating the contribution of each feature to its predictions. It helps to understand the behavior of the model by quantifying the contribution of each feature to the prediction result.

### Statistical analyses

Continuous variables are expressed as mean ± standard deviation, and the Shapiro-Wilk test was first used to test whether the variables followed a normal distribution. Student’s t-test or Mann-Whitney U test was then used. When comparing categorical variables, counts and percentages within each group were expressed as counts and percentages using the chi-square test or Fisher’s exact test. Statistical analysis was partially done by R version 4.4.2. *P* < 0.05 was considered statistically significant. The modeling and validation part of ML was done in Python version 3.9.

## Results

### Baseline analysis

In a comprehensive analysis of four NHANES cycles, 389 out of 2,467 subjects were identified as having MetS combined with low muscle mass. A total of 1,727 subjects were randomized into the training set and 740 subjects into the internal validation set. Male participants accounted for 50.1% of participants with low muscle mass and 47.7% of those without low muscle mass. Female participants accounted for 49.9% of those with low muscle mass and 52.3% of those without low muscle mass. Participants with low muscle mass exhibited wider waist circumferences (109 ± 14.2 cm vs. 115 ± 15.7 cm), higher ALP levels (74.0 ± 23.9 IU/L vs. 82.5 ± 27.1 IU/L), and neutrophil percentages (57.6 ± 8.92% vs. 59.4 ± 8.84%), than those without low muscle mass (all *p* < 0.05). Participants with low muscle mass had shorter thigh length (39.1 ± 3.56 cm vs. 35.8 ± 3.80 cm), shorter height (169 ± 9.30 cm vs. 159 ± 9.35 cm), lower PIR (2.44 ± 1.62 vs. 2.11 ± 1.56), and lower creatinine levels (139 ± 86.8 mg/L vs. 115 ± 71.5 mg/L) compared to those without low muscle mass (all *p* < 0.01). Other baseline information compared with participants without comorbidities is detailed in Table 1.

**Table 1:** Basic characteristics of participants in the NHANES 2011–2018.

Characteristic	Normal muscle mass	Low muscle mass risk	*P* value
Participants (n)	2078	389	
Gender (%)			0.418
Male	992 (47.7)	195 (50.1)	
Female	1086 (52.3)	194 (49.9)	
Age (years)	44.6 ± 10.5	46.8 ± 10.5	<0.001
Race (%)			<0.001
Non-Hispanic White	743 (35.8)	111 (28.5)	
Non-Hispanic Black	526 (25.3)	28 (7.2)	
Mexican American	301 (14.5)	135 (34.7)	
Other Race	299 (14.4)	45 (11.6)	
Other Hispanic	209 (10.1)	70 (18.0)	
Education (%)			<0.001
Below high school	415 (20.0)	132 (33.9)	
High school	465 (22.4)	101 (26.0)	
Above high school	1198 (57.7)	156 (40.1)	
Smoking (%)			0.106
Never smoker	1127 (54.2)	223 (57.3)	
Former smoker	425 (20.5)	87 (22.4)	
Current smoker	526 (25.3)	79 (20.3)	
PIR	2.44 ± 1.62	2.11 ± 1.56	<0.001
BMI (kg/m²)	33.1 ± 6.41	36.6 ± 7.20	<0.001
Diabetes (%)			<0.001
No	1346 (64.8)	196 (50.4)	
Yes	732 (35.2)	193 (49.6)	
Weight (kg)	94.8 ± 20.8	93.9 ± 23.8	0.075
Height (cm)	169 ± 9.30	159 ± 9.35	<0.001
Waist (cm)	109 ± 14.2	115 ± 15.7	<0.001
Upper arm length (cm)	38.0 ± 2.65	35.9 ± 2.76	<0.001
Arm circumference (cm)	36.3 ± 4.73	36.9 ± 5.08	0.046
Thigh length (cm)	39.1 ± 3.56	35.8 ± 3.80	<0.001
TC (mg/dL)	198 ± 46.5	198 ± 42.2	0.678
TG (mg/dL)	222 ± 232	213 ± 193	0.73
FPG (mg/dL)	125 ± 53.4	136 ± 64.2	<0.001
HbA1c (%)	6.22 ± 1.59	6.50 ± 1.66	<0.001
LDL (mg/dL)	111 ± 41.7	112 ± 38.7	0.456
HDL (mg/dL)	43.6 ± 11.8	44.0 ± 11.8	0.494
RBC (10¹²/L)	4.81 ± 0.498	4.87 ± 0.519	0.062
WBC (10⁹/L)	7.65 ± 2.20	8.21 ± 2.20	<0.001
PLT (10⁹/L)	251 ± 65.1	259 ± 72.6	0.032
HGB (g/dL)	14.2 ± 1.61	14.3 ± 1.59	0.219
ALT (U/L)	30.0 ± 21.8	33.0 ± 24.4	<0.001
AST (U/L)	26.8 ± 25.4	27.8 ± 18.7	0.003
GGT (IU/L)	37.1 ± 50.9	43.6 ± 57.6	<0.001
TBIL (mg/dL)	0.569 ± 0.285	0.540 ± 0.256	0.039
ALP (IU/L)	74.0 ± 23.9	82.5 ± 27.1	<0.001
ALB (mg/dL)	79.2 ± 487	66.7 ± 238	0.431
GLOB (g/L)	30.1 ± 4.83	30.7 ± 4.13	0.002
UA (umol/L)	343 ± 88.2	338 ± 88.2	0.329
BUN (mg/dL)	13.1 ± 5.50	13.4 ± 4.53	0.094
Cr (mg/L)	139 ± 86.8	115 ± 71.5	<0.001
LYM (10⁹/L)	2.33 ± 0.792	2.40 ± 0.730	0.05
MON (10⁹/L)	0.571 ± 0.192	0.589 ± 0.189	0.078
NEU (%)	57.6 ± 8.92	59.4 ± 8.84	<0.001
EOS (%)	2.87 ± 1.97	2.70 ± 2.08	0.01
BAS (%)	0.761 ± 0.406	0.740 ± 0.444	0.118
SBP (mmHg)	128 ± 16.2	129 ± 16.8	0.132
DBP (mmHg)	76.3 ± 12.2	75.5 ± 12.3	0.27
LDH (IU/L)	135 ± 33.7	137 ± 30.6	0.169
Calcium (mmol/L)	2.34 ± 0.093	2.33 ± 0.091	0.051
Iron (umol/L)	14.3 ± 6.22	14.1 ± 6.16	0.492
Sodium (mmol/L)	139 ± 2.46	139 ± 2.60	0.579
Potassium (mmol/L)	3.98 ± 0.346	3.98 ± 0.382	0.857
25OHD2 + 25OHD3 (nmol/L)	60.1 ± 25.1	59.2 ± 24.4	0.421

### Feature selection

**Fig 2:**
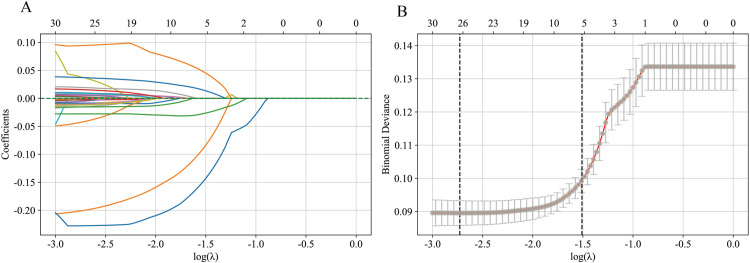
LASSO regression visualization plot. (A) This figure illustrates the regularization paths of coefficients for each feature in the LASSO regression model; (B) This figure shows the trend of binomial bias with λ and the optimal λ values determined by cross-validation.

LASSO regression can compress the coefficients of certain features to zero for feature selection by L1 regularization of the feature coefficients. We optimized the LASSO model using 5-fold cross-validation, and controlled the complexity of the model by introducing the regularization parameter lambda. Fig 2 is LASSO regression visualization plot. Finally, with the shrinkage parameter (lambda.1se), the five best predictive features, including height, gender, waist, thigh length, and ALP, were selected to be included in the ML model.

### Comparison of prediction models and subgroups analysis

**Fig 3:**
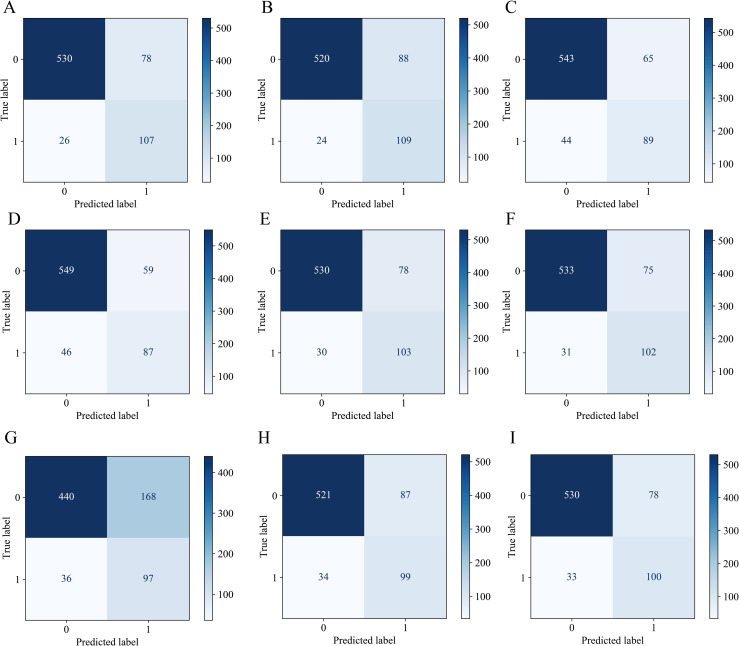
Confusion Matrix Diagram for 9 Models. (A) LR; (B) SVM; (C) RF; (D) XGB; (E) LGBM; (F) GBDT; (G) MLP; (H) DT; (I) CatBoost.

**Fig 4:**
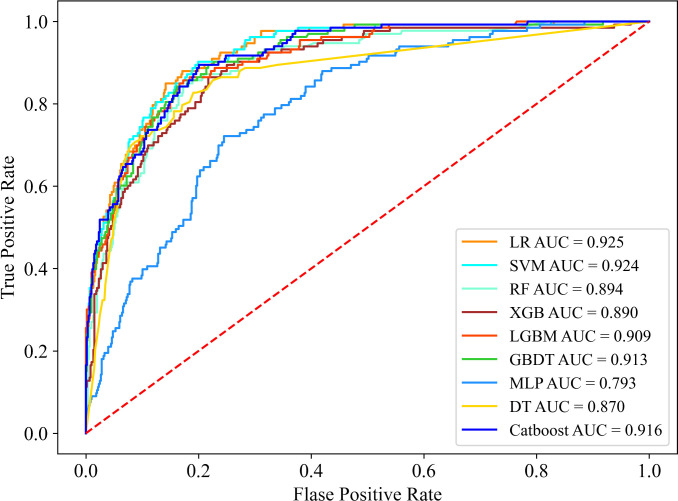
This figure presents the ROC curves and corresponding AUC values for various ML models. AUC values for each model: LR is 0.925 (95% CI: 0.904, 0.944), SVM is 0.924 (95% CI: 0.899, 0.945), RF is 0.894 (95% CI: 0.867, 0.923), XGB is 0.890 (95% CI: 0.859, 0.920), LGBM is 0.909 (95% CI: 0.8822, 0.9341), GBDT is 0.913 (95% CI: 0.888, 0.936), MLP is 0.793 (95% CI: 0.755, 0.828), DT is 0.870 (95% CI: 0.833, 0.904), CatBoost is 0.916 (95% CI: 0.891, 0.939).

Table 2 demonstrates the comprehensive performance metrics, including recall, precision, accuracy, F1-score, specificity, PPV, NPV, and AUC. The model confusion matrix is shown in Fig 3. Fig 4 shows the ROC curves. Considering all the metrics together, LR performs the best among the nine models in terms of performance, with the highest AUC (0.925), F1 score (0.673), and accuracy (0.860); ranked second in recall (0.805) and NPV (0.953); and showed moderate rankings in precision (0.578, tied for third) and specificity (0.872, tied for fourth). The ROC curves and the confusion matrix for the training set are presented in S1 Fig and S2 Fig, respectively. Moreover, formal statistical comparisons between models were conducted using the DeLong test. LR significantly outperformed the other models in terms of AUC. The details of these comparisons, including AUC differences, confidence intervals, and *p* values, are now reported in S2 Table.

**Table 2:** Model performance table for 9 models.

Model	Recall	Precision	Accuracy	F1	Specificity	PPV	NPV	AUC
LR	0.805	0.578	0.860	0.673	0.872	0.578	0.953	0.925
SVM	0.82	0.553	0.849	0.661	0.855	0.553	0.956	0.924
RF	0.669	0.578	0.853	0.62	0.893	0.578	0.925	0.894
XGB	0.654	0.596	0.858	0.624	0.903	0.596	0.923	0.890
LGBM	0.774	0.569	0.854	0.656	0.872	0.569	0.946	0.909
GBDT	0.767	0.576	0.857	0.658	0.877	0.576	0.945	0.913
MLP	0.729	0.366	0.725	0.487	0.724	0.366	0.924	0.793
DT	0.744	0.532	0.837	0.621	0.857	0.532	0.939	0.870
CatBoost	0.752	0.562	0.85	0.643	0.872	0.562	0.941	0.916

Based on the LR model, we conducted separate subgroup analyses by race and PIR to examine the effect size and statistical significance of key predictors within each group (Tables 3 and 4). According to official classifications published by the U.S. government, PIR was stratified into three levels: low (≤1.3), middle (1.3 to 3.5), and high (>3.5) [37]. In the subgroup analyses stratified by PIR, height, waist circumference, and sex remained consistently significant predictors of low muscle mass across all levels. Thigh length was a significant protective factor in the low and middle PIR groups, while ALP showed significance only in the low PIR group. Despite differences in statistical significance, the direction of associations for all predictors remained consistent across subgroups. Similarly, formal interaction tests between race and each predictor revealed no statistically significant interactions (all *p* > 0.05). These findings support the stability and fairness of the model across racial and socioeconomic subpopulations. Detailed odds ratios, 95% confidence intervals, and *p* values are provided in the S1 Table.

**Table 3:** Subgroup analysis of LR model performance stratified by PIR.

PIR group	Feature	OR	95% CI Lower	95% CI Upper	*P* value
High	Height	0.68	0.623	0.744	<0.001
High	Waist	1.101	1.072	1.13	<0.001
High	Thigh length	0.947	0.828	1.084	0.43
High	ALP	1.01	0.995	1.025	0.208
High	Gender	0.005	0.001	0.017	<0.001
Middle	Height	0.665	0.615	0.719	<0.001
Middle	Waist	1.109	1.084	1.135	<0.001
Middle	Thigh length	0.83	0.741	0.929	<0.001
Middle	ALP	1.01	1	1.02	0.05
Middle	Gender	0.001	0.0004	0.004	<0.001
Low	Height	0.743	0.702	0.786	<0.001
Low	Waist	1.083	1.063	1.105	<0.001
Low	Thigh length	0.899	0.818	0.987	<0.05
Low	ALP	1.01	1.002	1.019	<0.05
Low	Gender	0.013	0.006	0.028	<0.001

**Table 4:** Subgroup analysis of LR model performance stratified by race.

Race group	Feature	OR	95% CI Lower	95% CI Upper	*P* value
Non-Hispanic White	Height	0.693	0.644	0.746	<0.001
Non-Hispanic White	Waist	1.102	1.076	1.127	<0.001
Non-Hispanic White	Thigh length	0.932	0.835	1.041	0.215
Non-Hispanic White	ALP	1.007	0.997	1.017	0.151
Non-Hispanic White	Gender	0.005	0.002	0.015	<0.001
Non-Hispanic Black	Height	0.666	0.584	0.76	<0.001
Non-Hispanic Black	Waist	1.105	1.063	1.149	<0.001
Non-Hispanic Black	Thigh length	1.036	0.871	1.231	0.691
Non-Hispanic Black	ALP	1.008	0.984	1.032	0.541
Non-Hispanic Black	Gender	0.011	0.002	0.07	<0.001
Mexican American	Height	0.688	0.631	0.749	<0.001
Mexican American	Waist	1.111	1.081	1.142	<0.001
Mexican American	Thigh length	0.984	0.864	1.122	0.813
Mexican American	ALP	1.01	0.997	1.024	0.121
Mexican American	Gender	0.007	0.002	0.022	<0.001
Other Race	Height	0.587	0.496	0.695	<0.001
Other Race	Waist	1.128	1.076	1.182	<0.001
Other Race	Thigh length	0.984	0.808	1.199	0.875
Other Race	ALP	1.018	0.998	1.039	0.08
Other Race	Gender	0.001	0.0001	0.008	<0.001
Other Hispanic	Height	0.723	0.654	0.799	<0.001
Other Hispanic	Waist	1.098	1.059	1.139	<0.001
Other Hispanic	Thigh length	0.788	0.644	0.964	<0.05
Other Hispanic	ALP	1.008	0.994	1.023	0.267
Other Hispanic	Gender	0.005	0.001	0.024	<0.001

### Prediction results and feature interpretation based on SHAP values

**Fig 5:**
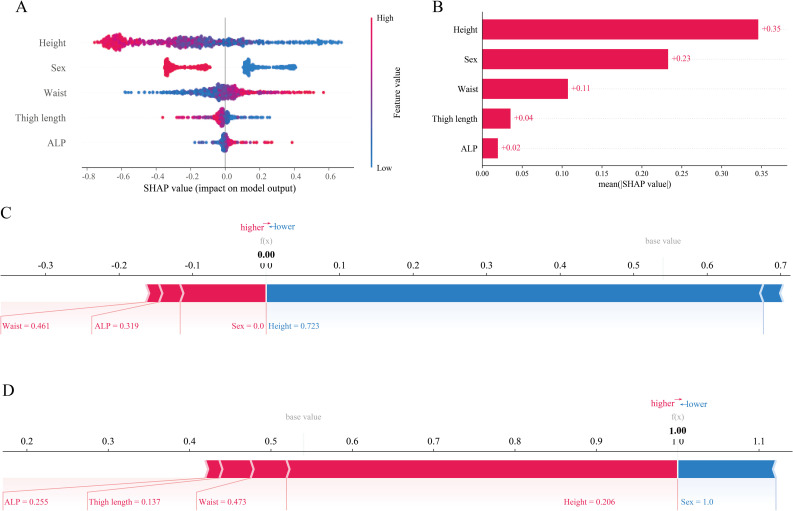
SHAP summary plot of features in the model. (A) A scatter plot of the SHAP values, showing the impact of each feature on the model output, the high and low feature values, and the direction of the impact. (B) A histogram of the mean SHAP values of the features, sorted by the mean impact of the features on the model output, showing the importance of each feature. (C) (D) Force plots of individual prediction instances, showing the specific contribution of each feature to the model prediction in a given instance.

**Fig 6:**
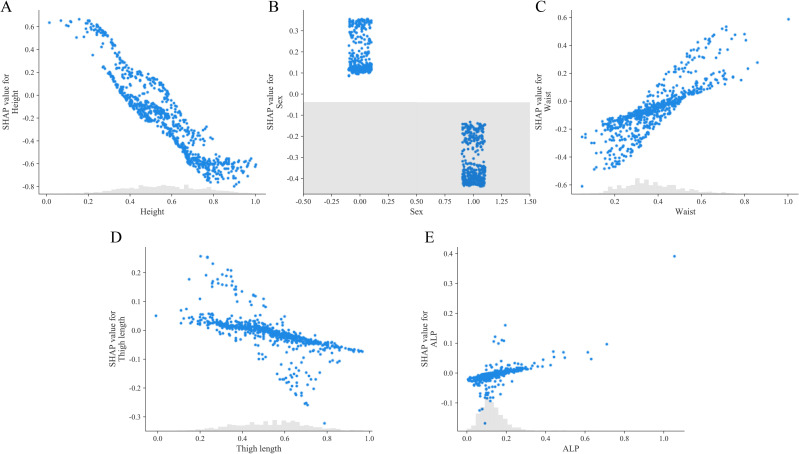
SHAP dependence plots. It shows the distribution pattern between SHAP values (vertical axis) and each characteristic value (horizontal axis).

Using readily available anthropometric and clinical biochemical indicators, five key risk factors were identified. Ranked by their SHAP values from highest to lowest, these were: height, gender, waist circumference, thigh length, and ALP. The significance and absolute SHAP values for each feature in the final LR model are shown in Fig 5. The SHAP plot highlights the key features associated with the risk of low muscle mass in the MetS, providing valuable insights into the development of personalized treatment strategies. The SHAP values on the horizontal axis reflect the extent to which the feature contributes to the model’s prediction of low muscle mass risk. Positive values increase the risk and negative values decrease the risk. The vertical axis shows the different features, with red points representing higher eigenvalues and blue points representing lower eigenvalues. The high red eigenvalues are mainly distributed on the right side of the zero line, indicating that the larger the eigenvalues are, the more they tend to push the disease prediction to the positive category. It can be seen that height contributes the most to the ML model. Fig 6 shows the SHAP dependence plots for each feature value.

### A web-based prediction tool

The tool can be found at: https://k4nkrgu39a3vjnzruudxwq.streamlit.app. The interface enables users to input relevant variables such as height, sex, waist circumference, thigh length, and ALP, and provides an individualized risk prediction. This information is provided in “Tool Interface and Usage Overview” (S1 File).

## Discussion

To the best of our knowledge, this is the inaugural study to explore the predictive modeling of low muscle mass in individuals with MetS. The pathogenesis of sarcopenia is complex, is associated with a poor prognosis, and is not yet fully recognized in individuals with MetS. As Caturano et al. have noted, these mechanisms highlight the importance of early-stage modeling in populations that are often underrepresented in clinical trials [38]. Simulation-based, data-driven tools may thus serve as an essential complement to conventional study designs for early risk identification.

### Anthropometric indicators

In clinical practice, routine anthropometric measurements serve as an effective early screening strategy for detecting muscle loss [39,40]. These indicators reflect both structural and compositional aspects of the body and are easily accessible in routine clinical assessments. This study indicated that height, waist circumference, and thigh length are strong predictors of low muscle mass in patients diagnosed with MetS.

Previous ML-based studies in diabetic and stroke populations have identified height as a key predictor of muscle mass loss [29,36]. This is in line with the results of the present study. A cohort study indicated that height loss has a detrimental effect on muscle mass and is associated with a higher incidence of sarcopenia and an elevated risk of mortality [41]. In MetS, chronic systemic inflammation and IR contribute to muscle protein breakdown and impaired muscle regeneration. Individuals with shorter stature may have a lower baseline skeletal muscle mass, making them more vulnerable to these catabolic effects. Additionally, altered hormonal profiles associated with shorter height, such as lower levels of growth hormone, insulin-like growth factor 1 (IGF-1), and anabolic sex steroids, can negatively impact muscle anabolism and repair processes.

Several surveys of Asians have also shown that patients with sarcopenia being significantly shorter than the normal group, supporting the role of stature as a clinically relevant risk factor. Moreover, height has been positively associated with skeletal muscle accrual during adolescence, which may reflect their parallel patterns of development [42]. In contrast, height loss in older adults has been identified as a simple surrogate marker for sarcopenia, further highlighting the association between stature and muscle mass [43].

Another finding in our study was that low muscle mass was associated with wider waist circumference. Wider waist circumference is often indicative of excessive accumulation of abdominal fat [44]. Visceral fat inhibits muscle function more than subcutaneous fat [45]. The relationship between waist circumference, a proxy for visceral fat, and sarcopenia is bidirectional. Abnormal lipid metabolism triggered by visceral fat accumulation exacerbates muscle damage through two parallel pathways. Specifically, free fatty acids (FFA) inhibits the tyrosine phosphorylation process of skeletal muscle insulin receptor substrate-1 (IRS-1) and blocks the PI3K/Akt signaling pathway, which reduces glucose uptake as well as myosin synthesis, leading to IR [46]. Meanwhile, adipokine imbalance interfered with JAK-STAT/AMPK signaling, further impairing muscle repair [47,48]. In addition, the decrease in muscle mass lowers the basal metabolic rate, which further promotes the accumulation of fat, creating a vicious cycle of “sarcopenic obesity”. This is a condition where there is a confluence of decreased muscle mass and increased body fat [49]. The degree of abdominal obesity in individuals with MetS was greater in those with sarcopenia compared to those without sarcopenia, which is consistent with previous studies [50].

An interesting finding of the study was that thigh length showed value in predicting low muscle mass in a young MetS population. Although its SHAP score was low, indicating a relatively weak predictive role in the model. In anthropometry, thigh length primarily corresponds to the anatomical length of the femur. Imaging-based investigations have shown that thigh muscle volume correlates with femoral length, suggesting that thigh length may indirectly reflect underlying muscle reserve [51]. The identification of thigh length as a predictor in our study is novel. Future research is required to further confirm the link between thigh length and risk factors for low muscle mass.

While they do not directly represent muscle mass or function, they have been shown to correlate with skeletal muscle volume in studies and may serve as surrogate markers in the absence of direct muscle assessment. Their predictive value in this study may reflect anthropometric and developmental influences on muscle distribution. However, the underlying mechanisms linking these variables to muscle loss remain unclear and warrant further investigation.

### Gender and age factors

Our study found that low muscle mass exhibits gender specific patterns and is more prevalent among males, which is consistent with certain epidemiological evidence. Xu et al. demonstrated that the mean ASM and appendicular lean mass values of adult males continued to decrease with age, while the mean ASM and total skeletal muscle mass values of females increased slightly [42]. Women are slightly less affected, with a 1.53-fold increase [10]. Similarly, a UK Biobank-based assessment indicated that adult men had a higher incidence of low muscle mass than adult women [52]. Although several studies have reported that the prevalence of sarcopenia is lower in women. Some research, however, the correlation between sex and the prevalence of sarcopenia was reversed. The prevalence of sarcopenia was found to be significantly greater among women compared to men in a cross-sectional study of 2,697 participants [53]. These discrepancies may partly reflect age and gender specific changes in muscle composition and hormone dynamics, including the age related decline of anabolic hormones such as testosterone and IGF-1 [37,38]. The influence of gender on low muscle mass remains a subject of debate. Future studies should expand the sample size and include a greater number of patients of different ages and genders for observation, which will facilitate the identification of risk factors for low muscle mass more.

### Biochemical markers

As a nonspecific serum biomarker, ALP serves an important function in bone metabolism and is commonly used in clinical evaluations [54]. However, its potential association with muscle loss and sarcopenia remains insufficiently explored and may involve complex inflammatory and metabolic mechanisms. One possible explanation is that elevated ALP levels indicate heightened oxidative stress and mitochondrial dysfunction in muscle, leading to accelerated muscle proteolysis [55].

Recent research has demonstrated an inverse relationship between serum ALP levels and muscle strength, particularly in middle-aged individuals. ALP was inversely correlated with grip strength in both men and women in a cross-sectional research of 3,514 participants; the correlation was stronger in the 40–59 age range [56]. Lee et al. found that ALP levels were more predictive of sarcopenia than white blood cell counts and were independently and positively associated with low muscle mass [57]. These findings support ALP as a indicator of muscle deterioration, consistent with our study’s results.

In conclusion, the risk of low muscle mass in patients with MetS is influenced by multidimensional factors, and shortened height, gender differences, abdominal obesity, elevated ALP, and shortened thigh length can be used as early warning signs. In this study, the ML model we developed, especially LR, demonstrated high predictive efficacy in predicting the risk of pre-sarcopenia in young MetS patients. A meta-analysis of 71 studies revealed that other ML models did not demonstrate superiority over LR in terms of performance in clinical prediction models [58].

### Limitations and future directions

Nevertheless, there are still several restrictions on this study. First, the use of a cross-sectional dataset such as NHANES restricts causal interpretation, as temporal relationships between predictors and outcomes cannot be established. Second, although we applied LASSO to mitigate redundancy, the model may still be susceptible to overfitting or selection bias. Third, the sample size was relatively limited, and external validation using large, independent, and longitudinal cohorts is necessary to enhance generalizability and clinical applicability. Future studies are needed to externally validate the model, incorporate functional performance measures, and assess its generalizability across diverse populations. Moreover, the application of AI and machine learning in low-and middle-income countries (LMICs) faces unique challenges, including limited infrastructure, digital capacity, and affordability [59]. To support global health equity, future research should prioritize scalable, cost-effective, and locally adaptable solutions.

## Conclusion

In this study, nine different predictive models were constructed using the NHANES database to identify factors associated with pre-sarcopenia in a MetS population. Common anthropometric assessment indicators and readily available laboratory tests were used in the model, finalizing a total of five indicators. Height, sex, and waist circumference contributed substantially to the model’s predictive performance, whereas thigh length and ALP made relatively smaller contributions. These findings may contribute to the early detection and timely intervention of muscle mass loss in individuals with MetS.

## Supporting information

S1 Fig ROC curves and corresponding AUC values for different models in the training set. (A) LR; (B) SVM; (C) RF; (D) XGB; (E) LGBM; (F) GBDT; (G) MLP; (H) DT; (I) CatBoost.
(TIF)

S2 Fig Confusion matrix plots for the 9 models in the training set. (A) LR; (B) SVM; (C) RF; (D) XGB; (E) LGBM; (F) GBDT; (G) MLP; (H) DT; (I) CatBoost.
(TIF)

S1 Table Interaction analysis of race subgroups in the LR model. (PDF)

S2 Table DeLong test for AUCs. (PDF)

S1 File Tool Interface and Usage Overview. (PDF)
