# Supplementary figures and images for: Construct prediction models for low muscle mass with metabolic syndrome using machine learning
Source: PLoS One. 2025 Sep 9;20(9):e0331925. doi: 10.1371/journal.pone.0331925 (PMC12419592; doi:10.1371/journal.pone.0331925)

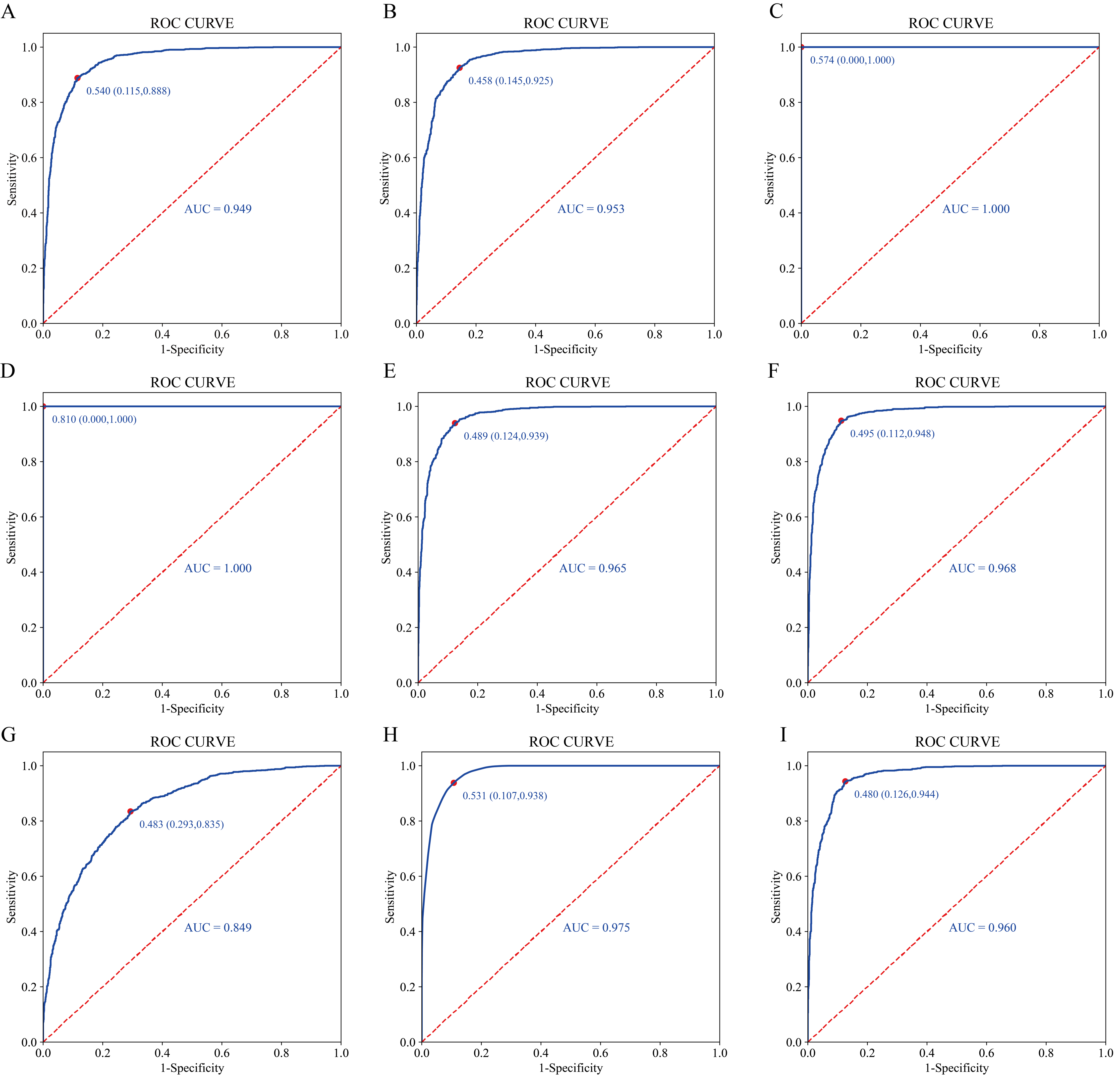

Supplement: S1 Fig — (A) LR; (B) SVM; (C) RF; (D) XGB; (E) LGBM; (F) GBDT; (G) MLP; (H) DT; (I) CatBoost. (TIF) [file pone.0331925.s001.tif]

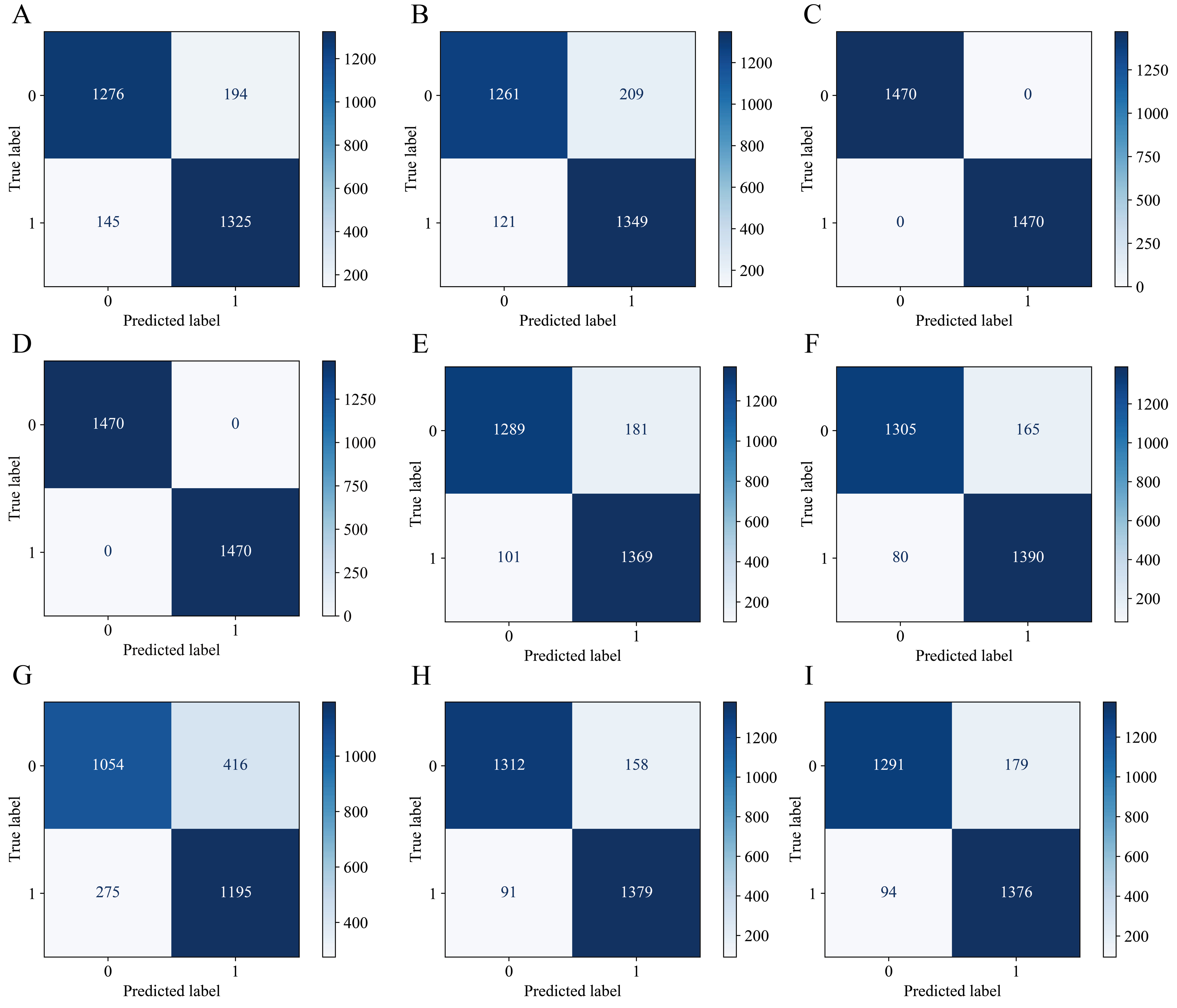

Supplement: S2 Fig — (A) LR; (B) SVM; (C) RF; (D) XGB; (E) LGBM; (F) GBDT; (G) MLP; (H) DT; (I) CatBoost. (TIF) [file pone.0331925.s002.tif]
