# Supplementary material for: Construct prediction models for low muscle mass with metabolic syndrome using machine learning
Source: PLoS One. 2025 Sep 9;20(9):e0331925. doi: 10.1371/journal.pone.0331925 (PMC12419592; doi:10.1371/journal.pone.0331925)
Supplement: S1 Table — (PDF) [file pone.0331925.s003.pdf]

S1 Table. Interaction analysis of race subgroups in the LR model.

Feature	Interaction Term	OR	95% CI Lower	95% CI Upper	*P* value
Height	Height:Race	0.999	0.989	1.009	0.848
Waist	Waist:Race	0.998	0.992	1.003	0.377
Thigh length	Thigh length:Race	1.002	0.977	1.028	0.868
ALP	ALP:Race	1.002	1.000	1.005	0.082
Gender	Gender:Race	0.934	0.802	1.089	0.386
