# Supplementary material for: Construct prediction models for low muscle mass with metabolic syndrome using machine learning
Source: PLoS One. 2025 Sep 9;20(9):e0331925. doi: 10.1371/journal.pone.0331925 (PMC12419592; doi:10.1371/journal.pone.0331925)
Supplement: S2 Table — (PDF) [file pone.0331925.s004.pdf]

S2 Table. DeLong test for AUCs.

Model 1	Model 2	AUC 1	AUC 2	AUC Diff	Z score	*P* value	95% CI Lower	95% CI Upper
LR	MLP	0.925	0.793	0.132	6.904	<0.001	0.094	0.169
SVM	MLP	0.924	0.793	0.131	6.836	<0.001	0.093	0.168
MLP	CatBoost	0.793	0.916	-0.123	-6.55	<0.001	-0.159	-0.086
GBDT	MLP	0.913	0.793	0.12	6.286	<0.001	0.082	0.157
LGBM	MLP	0.909	0.793	0.116	5.826	<0.001	0.077	0.155
RF	MLP	0.894	0.793	0.101	5.204	<0.001	0.063	0.139
XGB	MLP	0.89	0.793	0.097	4.992	<0.001	0.059	0.135
SVM	DT	0.924	0.87	0.054	3.987	<0.001	0.027	0.08
SVM	XGB	0.924	0.89	0.034	3.968	<0.001	0.017	0.05
LR	DT	0.925	0.87	0.055	3.959	<0.001	0.028	0.082
LR	XGB	0.925	0.89	0.034	3.929	<0.001	0.017	0.051
SVM	RF	0.924	0.894	0.03	3.806	<0.001	0.014	0.045
DT	CatBoost	0.87	0.916	-0.046	-3.592	<0.001	-0.071	-0.021
LR	RF	0.925	0.894	0.031	3.55	<0.001	0.014	0.047
XGB	CatBoost	0.89	0.916	-0.025	-3.387	0.001	-0.04	-0.011
LGBM	DT	0.909	0.87	0.039	3.33	0.001	0.016	0.062
GBDT	DT	0.913	0.87	0.043	3.299	0.001	0.017	0.068
MLP	DT	0.793	0.87	-0.077	-3.188	0.001	-0.124	-0.03
RF	CatBoost	0.894	0.916	-0.022	-3.129	0.002	-0.035	-0.008
XGB	GBDT	0.89	0.913	-0.022	-2.992	0.003	-0.037	-0.008

SVM	LGBM	0.924	0.909	0.015	2.835	0.005	0.004	0.025
XGB	LGBM	0.89	0.909	-0.019	-2.638	0.008	-0.033	-0.005
LR	LGBM	0.925	0.909	0.015	2.615	0.009	0.004	0.027
SVM	GBDT	0.924	0.913	0.011	2.61	0.009	0.003	0.02
RF	GBDT	0.894	0.913	-0.018	-2.544	0.011	-0.033	-0.004
LR	GBDT	0.925	0.913	0.012	2.46	0.014	0.002	0.022
SVM	CatBoost	0.924	0.916	0.008	2.207	0.027	0.001	0.015
LR	CatBoost	0.925	0.916	0.009	2.154	0.031	0.001	0.017
RF	LGBM	0.894	0.909	-0.015	-2.095	0.036	-0.029	-0.001
RF	DT	0.894	0.87	0.024	1.794	0.073	-0.002	0.05
LGBM	CatBoost	0.909	0.916	-0.006	-1.554	0.12	-0.014	0.002
XGB	DT	0.89	0.87	0.02	1.489	0.136	-0.006	0.047
GBDT	CatBoost	0.913	0.916	-0.003	-0.866	0.387	-0.01	0.004
LGBM	GBDT	0.909	0.913	-0.003	-0.847	0.397	-0.011	0.004
RF	XGB	0.894	0.89	0.004	0.556	0.578	-0.01	0.017
LR	SVM	0.925	0.924	0.001	0.352	0.725	-0.004	0.005
