# Supplementary material for: Construct prediction models for low muscle mass with metabolic syndrome using machine learning
Source: PLoS One. 2025 Sep 9;20(9):e0331925. doi: 10.1371/journal.pone.0331925 (PMC12419592; doi:10.1371/journal.pone.0331925)
Supplement: S1 File — (PDF) [file pone.0331925.s005.pdf]

S1 File. Tool Interface and Usage Overview

We developed a user-friendly web-based interface to facilitate prediction of syndrome risk based on physiological parameters. The interface allows users to input five variables: Height (cm), Waist circumference (cm), Thigh Length (cm), ALP (U/L), and Sex (Male/Female). A "Predict" button triggers the underlying model to return the probability of a positive diagnosis.

On the left panel, a brief user guide is provided, including reference ranges for each physiological indicator (e.g., normal adult height: 150–200 cm; ALP: 40–160 U/L).

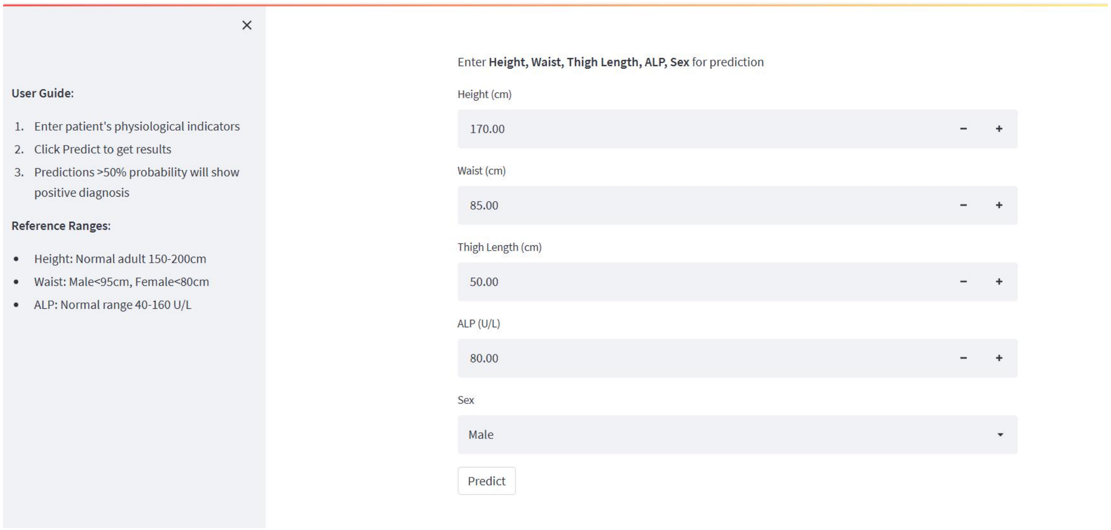

This tool is designed for use by clinicians or researchers as a lightweight screening aid. For instance, after inputting a patient's basic measurements, the clinician can receive a prediction result in under one second. Figure shows a snapshot of the interface.

Example Use Case:

A male patient with Height = 170 cm, Waist = 85 cm, Thigh Length = 50 cm, ALP = 80 U/L is entered into the system. Upon clicking "Predict", the tool returns a risk probability.
